# Editorial to the Special Issue “Gulliver in the Country of Lilliput: An Interplay of Noncovalent Interactions”

**DOI:** 10.3390/molecules26010158

**Published:** 2020-12-31

**Authors:** Ilya G. Shenderovich

**Affiliations:** Institute of Organic Chemistry, Faculty of Chemistry and Pharmacy, University of Regensburg, Universitaetstrasse 31, 93053 Regensburg, Germany; Ilya.Shenderovich@ur.de

Noncovalent interactions allow our world to exist. Their study remains vital to the progress of chemistry and chemical physics. This topic has been specifically addressed in a number of the past and present Special Issues of *Molecules*, and almost every publication touches on the subject of noncovalent interactions in one way or another.

The overarching goal of this Special Issue was to bring together publications that consider effects caused by an interplay of noncovalent interactions. A common case is the situation when there is one dominant interaction that determines the structure of the molecular system, and a large number of weaker interactions that are forced to adapt to this structure. Although it is clear that this “Gulliver in the Country of Lilliput” model is only a rough approximation, this view implicitly prompts the assumption that the net effect of weak interactions is negligible, at least on the structure of the system, since multiple small contributions can cancel each other out. In some cases, this may turn out to be true. However, since the total strength of the “Lilliputians” can exceed that of the “Gulliver”, a priori conclusions can lose their predictive power. The dominant interaction can be strong and protected from any direct competition, as in the case of the proton-bound homodimer of pyridine, but the geometry of this complex still differs in the gas, solution, and solid phases [1]. Similarly, the result of competition between two strong interactions can be determined by weak interactions [2]. The current challenge is to learn to incorporate multiple competing interactions into effective working models.

The contributions in this Special Issue can be grouped into three thematic areas: (i) specific properties of selected interactions evaluated for bi- or trimolecular complexes, [3,4,5,6] (ii) their role in the crystal packing [7,8], chemical [9,10] and enzymatic [11] reactions as well as (iii) manifestations of competing noncovalent interactions in solution [12] and into porous materials [13].

The experimentally challenging study of Suhm et al. [3] analyses the docking preference of alcohols between the two nonequivalent lone electron pairs of the carbonyl group in pinacolone using supersonic jet expansions of 1:1 solvate complexes. The result of an interplay between the nonequivalence of the lone electron pairs and distant London dispersion and Pauli repulsion was modulated by the size of the alkyl group of the alcohol. The obtained experimental results serve as extremely high level benchmarks for verifying the accuracy of theoretical methods. Some of these methods were tested. It is suggested to note the importance of London dispersion for structure and stability of molecular aggregates [14].

The paper by Szatylowicz et al. [4] describes the effect of substituents on the energy of specific noncovalent interactions in adenine-based bimolecular complexes and the aromaticity of the partners. Understanding these effects is essential for effective control of acid-base interactions in biochemistry, where stronger does not always mean better. Special attention was payed to the comparison of the energies obtained using different models. The reader may be interested in further reading on this subject [15].

The contribution by Filarowski et al. [5] reports on the balance of repulsive and attractive intramolecular interactions between adjacent carboxyl groups in selectively substituted phthalic acids, the dependence of this balance on intramolecular steric crowding, and the effect of these intramolecular properties on intermolecular interactions of these carboxyl groups. This study represents a combination of the Infrared, Raman, Nuclear Magnetic Resonance (NMR), and Incoherent Inelastic Neutron Scattering spectroscopies and the Car–Parrinello Molecular Dynamics and Density Functional Theory calculations. The structural and energetic parameters of the intra- and intermolecular interactions were estimated for the gas, liquid, and solid phases. Note that despite the originality of the outcomes arising from neutron scattering, the number of molecular systems studied by these methods is limited due to the complexity of the experimental equipment required [16,17].

The paper by Tolstoy et al. [6] describes halogen-bonded complexes formed by trimethylphosphine oxide with 128 different halogen donors of various classes. Correlations between the energetic, geometric and spectral properties of these complexes were established and summarized. These correlations make it possible to estimate the energy and geometry of a given halogen bond from the corresponding experimentally measured spectral parameter. Both the halogen bonding and the acceptor properties of the P=O moiety [18] are of considerable current interest. Therefore, the reported correlations can be very useful.

Vener et al. [7] describe how the calculated structural and spectral parameters of two component crystals of organic salts depend on various parameters of the theoretical approximation used. The experimental parameters of such systems cannot be correctly reproduced using the approximation of small molecular clusters. Using the periodic density functional theory is a reasonable compromise that allows the parameters of complex multicomponent pharmaceuticals to be accurately predicted [19].

The paper by Nenajdenko, Tskhovrebov et al. [8] demonstrates that halogen-halogen interactions play a critical role in self-assembly of highly polarizable molecules in crystals. A series of novel halogenated aromatic dichlorodiazadienes were prepared and characterized using X-ray diffraction and Bader’s Theory of Atoms in Molecules. Although halogen-halogen interactions are not strong, they can be a tool for fine-tuning the crystal structure [20].

The review by Grabowski [9] highlights the role of noncovalent interactions as a preliminary stage of chemical reactions. Hydrogen bonding assisted proton transfer, halogen bonding in solution, molecular hydrogen elimination via a dihydrogen bond, the intramolecular conformational effect of triel bonds, and tetrel bonds involving S_N_2 reactions were considered. Note that these short-lived interactions can both facilitate and hinder other steps [21,22]. For example, Ke and Lin [10] report on the catalytic effect of hydrogen bonding on the methanol steam reforming reaction on a metal surface. This and other publications on this topic may be directly in demand for practical use [23].

Vianello et al. [11] demonstrate how small alterations in weak interactions can cause significant changes in biological activity. Their molecular dynamic simulations correctly reproduce experimental data on the binding energy of histamine within the H_2_ receptor and its change caused by deuteration. The fact that deuteration can affect the kinetics of a chemical reaction [24] and result in measurable structural and spectral changes [25] is well known. However, in the paper at hand, the authors were able to identify the mechanism responsible for these changes.

The paper by Shenderovich and Denisov [12] presents an advanced approach to implicitly accounting for the solvent effect. In this adduct-under-field approach, the solvent effect is simulated using an external electric field. It was shown that solute–solvent interactions remarkably affect the geometry of acid-base complexes even if the active sites of these complexes are not accessible for solvent molecules. Note that this approach is applicable to many other molecular systems in solution and in crystal form [26,27].

The review of Buntkowsky and Vogel [13] describes current trends and perspectives in the study of guest molecules in porous silica materials employing solid-state NMR techniques with particular attention to the effect of an interplay between guest–guest and guest–host interactions. It is shown that such interactions can radically change the physicochemical properties of these systems. Solid-state NMR and relaxometry are among the most effective analytical tools in this area of materials chemistry. They can be applied for probing structure or dynamics of materials themselves as well as the behavior of incorporated guests [28,29].
